# Correction: Tokumaru et al. High Expression of microRNA-143 Is Associated with Favorable Tumor Immune Microenvironment and Better Survival in Estrogen Receptor Positive Breast Cancer. *Int. J. Mol. Sci.* 2020, *21*, 3213

**DOI:** 10.3390/ijms27156863

**Published:** 2026-07-31

**Authors:** Yoshihisa Tokumaru, Mariko Asaoka, Masanori Oshi, Eriko Katsuta, Li Yan, Sumana Narayanan, Nobuhiko Sugito, Nobuhisa Matsuhashi, Manabu Futamura, Yukihiro Akao, Kazuhiro Yoshida, Kazuaki Takabe

**Affiliations:** 1 Breast Surgery, Department of Surgical Oncology, Roswell Park Comprehensive Cancer Center, Buffalo, NY 14263, USA; yoshitoku1090@gmail.com (Y.T.); 590mariko@gmail.com (M.A.); masanori.oshi@roswellpark.org (M.O.); eriko.katsuta@roswellpark.org (E.K.); 2 Department of Surgical Oncology, Graduate School of Medicine, Gifu University, 1-1 Yanagido, Gifu 501-1194, Japan; nobuhisa@gifu-u.ac.jp (N.M.); mfutamur@gifu-u.ac.jp (M.F.); kyoshida@gifu-u.ac.jp (K.Y.); 3 Department of Breast Surgery and Oncology, Tokyo Medical University, Tokyo 160-8402, Japan; 4 Department of Surgery, Yokohama City University, Yokohama 236-0004, Japan; 5 Department of Biostatistics & Bioinformatics, Roswell Park Cancer Institute, Buffalo, NY 14263, USA; li.yan@roswellpark.org; 6 Department of Surgical Oncology, Mount Sinai Medical Center, Miami Beach, FL 33140, USA; narayanan.sumana@gmail.com; 7 United Graduate School of Drug and Medical Information Sciences, Gifu University, Gifu 501-1194, Japan; nsugito@gifu-u.ac.jp (N.S.); yakao@gifu-u.ac.jp (Y.A.); 8 Department of Surgery, University at Buffalo Jacobs School of Medicine and Biomedical Sciences, The State University of New York, Buffalo, NY 14203, USA; 9 Department of Surgery, Niigata University Graduate School of Medical and Dental Sciences, Niigata 951-8510, Japan; 10 Department of Breast Surgery, Fukushima Medical University, Fukushima 960-1295, Japan

The following reference [8] has been retracted and has therefore been removed from the original publication [1]:

8. He, Z.; Yi, J.; Liu, X.; Chen, J.; Han, S.; Jin, L.; Chen, L.; Song, H. MiR-143-3p functions as a tumor suppressor by regulating cell proliferation, invasion and epithelial–mesenchymal transition by targeting QKI-5 in esophageal squamous cell carcinoma. *Mol. Cancer* **2016**, *15*, 51.

In addition, reference [14] has been removed from the original publication:

14 Yan, X.; Chen, X.; Liang, H.; Deng, T.; Chen, W.; Zhang, S.; Liu, M.; Gao, X.; Liu, Y.; Zhao, C.; et al. miR-143 and miR-145 synergistically regulate ERBB3 to suppress cell proliferation and invasion in breast cancer. *Mol. Cancer* **2014**, *13*, 220.

Following these changes, the order of some references has been adjusted accordingly. The authors state that the scientific conclusions are unaffected. This correction was approved by the Academic Editor. The original publication has been updated.
