# Supplementary material for: High Expression of microRNA-143 is Associated with Favorable Tumor Immune Microenvironment and Better Survival in Estrogen Receptor Positive Breast Cancer
Source: Int J Mol Sci. 2020 May 1;21(9):3213. doi: 10.3390/ijms21093213 (PMC7246786; doi:10.3390/ijms21093213)
Supplement: Supplementary file 1 [file ijms-21-03213-s001.pdf]

Supplementary Materials

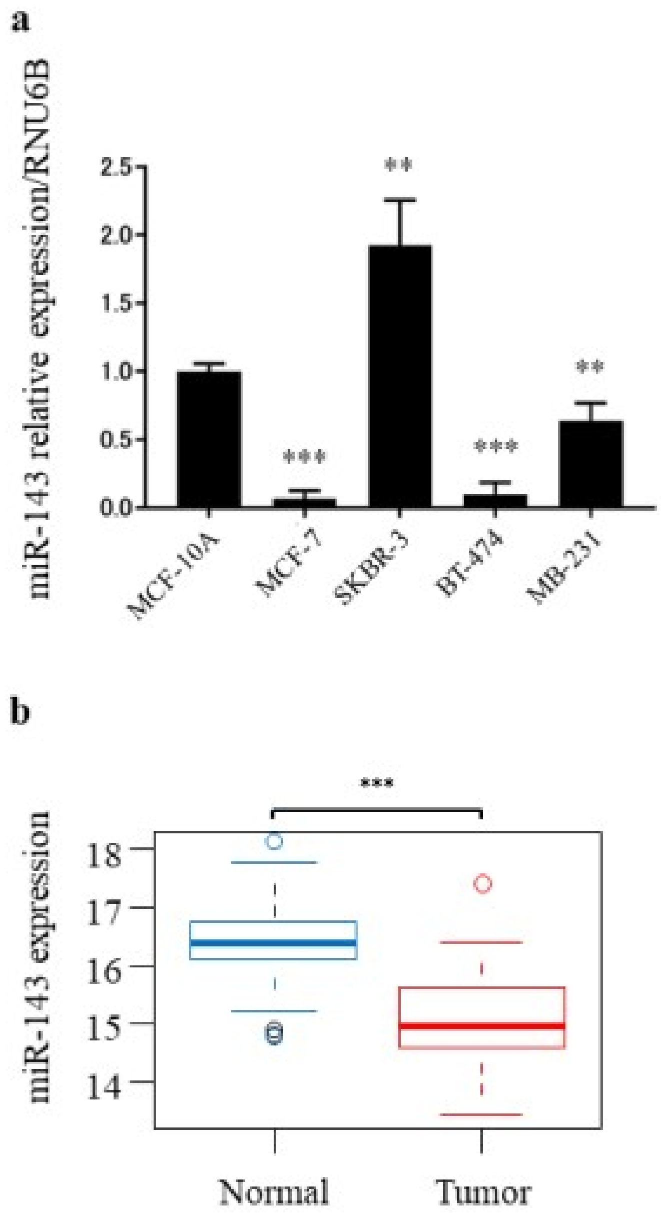

Figure S1. Downregulation of miR-143 in breast cancer cell lines when compared with normal epithelial cell line (**a**). Downregulation of miR-143 with tumor samples compared with normal tissue in clinical samples (**b**). ** $p < 0.01$; *** $p < 0.001$.

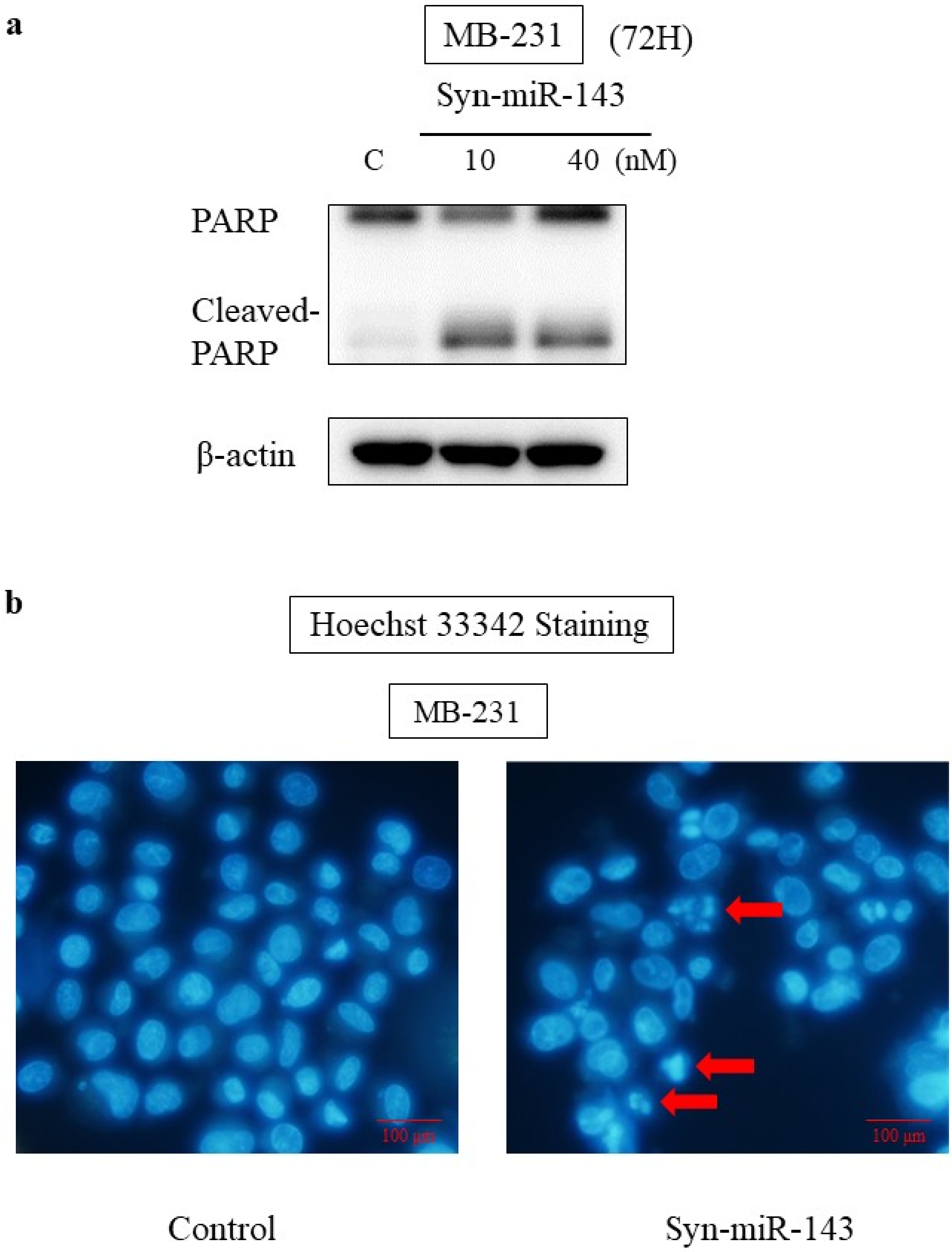

Figure S2. Western blot analysis of PARP at 72 h after transfection with control RNA (40 nM) or syn-miR-143 (10 nM, 40 nM) to MB-231 cells (**a**). Hoechst 33342 staining of MB-231 cells at 96 h after transfection with control RNA (10 nM) or syn-miR-143 (10 nM) (**b**).

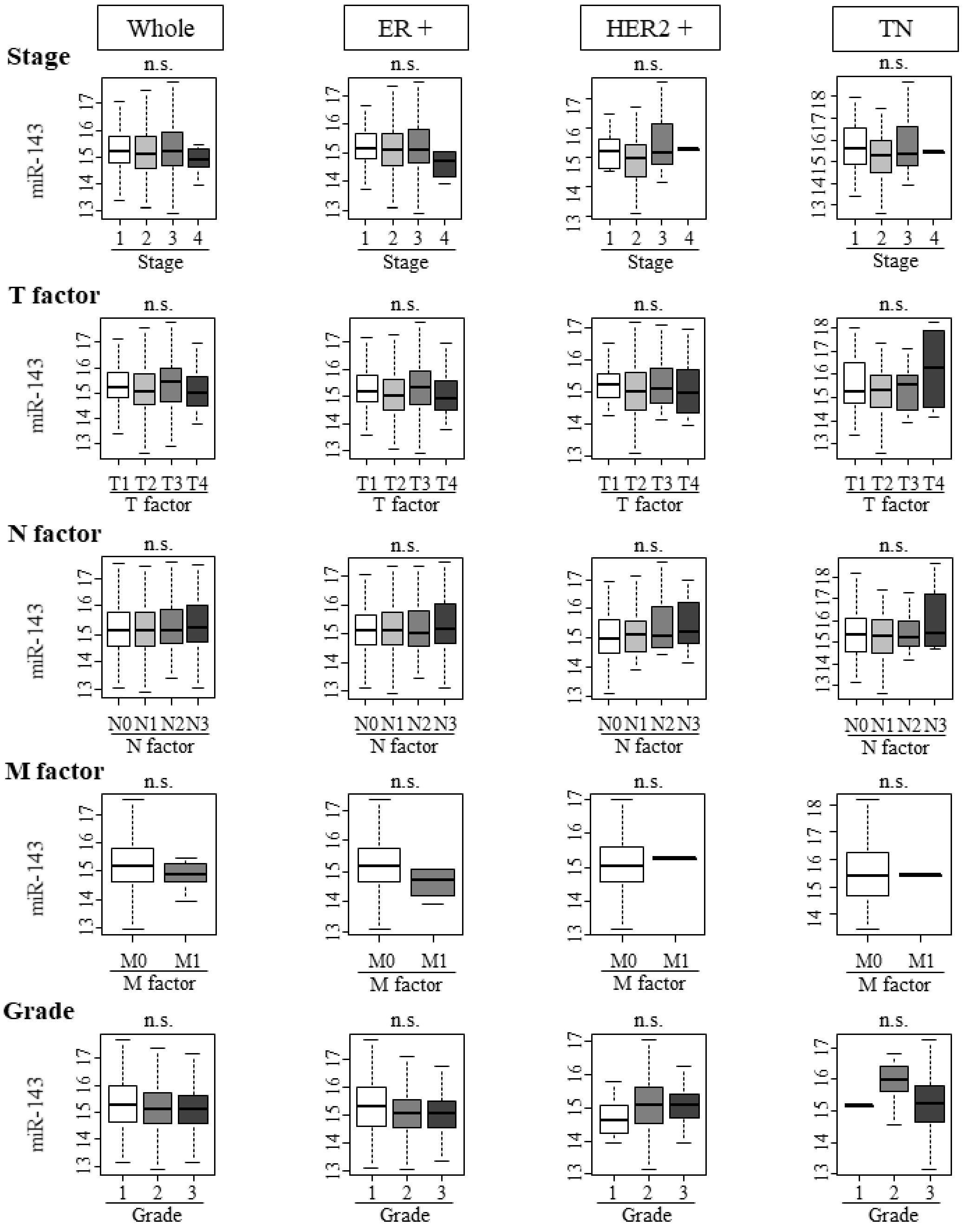

Figure S3. The association between miR-143 expression and clinical stage and histological grades. The association between miR-143 expression and patient clinicopathological features between miR-143 high and miR-143 low group in whole groups and different subtypes. n.s., not statistically significant. ER+, estrogen receptor positive; HER2, human epidermal growth factor receptor 2; TN, triple negative.

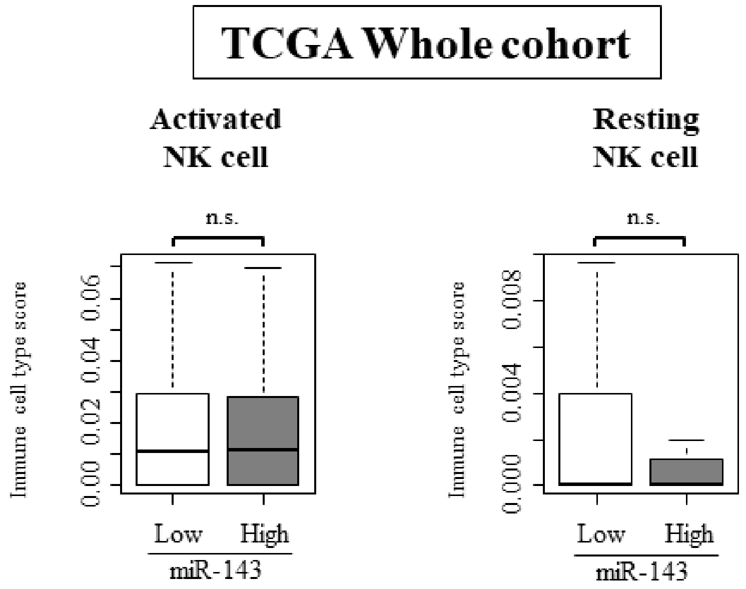

Figure S4. Infiltration of NK cells did not demonstrate the significant difference between miR-143 high and low tumors with TCGA whole cohort. n.s., not statistically significant. NK cell, Natural killer cell.

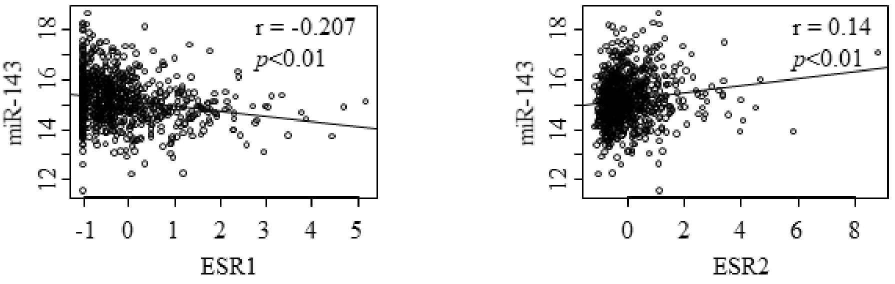

Figure S5. Assessing correlation between miR-143 and ESR1 or ESR2 with Pearson correlation analysis on TCGA cohort.
